# Model organisms and systems in neuroethology: one hundred years of history and a look into the future

**DOI:** 10.1007/s00359-023-01685-z

**Published:** 2024-01-16

**Authors:** Hermann Wagner, Martin Egelhaaf, Catherine Carr

**Affiliations:** 1 Institute of Biology II, RWTH Aachen University, 52074 Aachen, Germany; 2 Department of Neurobiology, Bielefeld University, Bielefeld, Germany; 3 Department of Biology, University of Maryland at College Park, College Park, USA

**Keywords:** Barn owl, Fly, Electric fish, Motion vision, Sound localization, Electrolocation

## Abstract

The Journal of Comparative Physiology lived up to its name in the last 100 years by including more than 1500 different taxa in almost 10,000 publications. Seventeen phyla of the animal kingdom were represented. The honeybee (*Apis mellifera*) is the taxon with most publications, followed by locust (*Locusta migratoria*), crayfishes (*Cambarus* spp.), and fruitfly (*Drosophila melanogaster*). The representation of species in this journal in the past, thus, differs much from the 13 model systems as named by the National Institutes of Health (USA). We mention major accomplishments of research on species with specific adaptations, specialist animals, for example, the quantitative description of the processes underlying the axon potential in squid (*Loligo forbesii*) and the isolation of the first receptor channel in the electric eel (*Electrophorus electricus*) and electric ray (*Torpedo* spp.). Future neuroethological work should make the recent genetic and technological developments available for specialist animals. There are many research questions left that may be answered with high yield in specialists and some questions that can only be answered in specialists. Moreover, the adaptations of animals that occupy specific ecological niches often lend themselves to biomimetic applications. We go into some depth in explaining our thoughts in the research of motion vision in insects, sound localization in barn owls, and electroreception in weakly electric fish.

**Supplementary Information:**

The online version contains supplementary material available at 10.1007/s00359-023-01685-z.

## Introduction

Neuroethology is a discipline grounded in the behavioral and neural adaptations provided by evolution. This approach has been very successful. The study of a broad variety of species has been challenged by concentration of the research on a few species, also called model systems, by most researchers and the development and application of new techniques in these few species. This is a serious challenge for comparative studies, and we ask what are the future perspectives of the neuroethological approach?^1^ The term "comparative" has been used by critics to mean that researchers choose just another species, because they do not have novel ideas. This is not only unjust, but also denies the richness of information embedded in different solutions to an evolutionary challenge in closely related taxa. As biologists, we believe that all questions should be viewed under the viewpoint of evolution. For example, if we want to understand human diseases, we need to consider evolution. This has become clear even to the public in the course of the COVID pandemic when the awareness rose that zoonoses are a major source of human diseases. Neuroethologists, who try to understand behavior and neural processes, need to take into account the evolution of the brain and its circuits. We start with an analysis of the history of the publications in this journal. This historical overview will provide insights not only into what was important so far, but also hint what directions may be the most significant in future.

### Analysis of past publications

Biology hides many treasures. These manifest themselves as specific adaptations acquired in the course of evolution. The almost 10,000 publications that appeared in the Journal of Comparative Physiology A (formerly Zeitschrift für vergleichende Physiologie) since 1924 contributed a lot to unravel and understand these adaptations (Tables S1 and S2). The authors examined more than 1500 taxa^2^ from 17 phyla of the animal kingdom, but also included some plant species and one study on a bacterium (Table 1).

**Table 1:** Distribution of taxa

Bacteria	1		Plathelminthes	26		Onychophora	1
Plantae	8		Bryozoa	3		Arthropoda	4378
Protista	89		Annelida	198		Echinodermata	23
Ctenophora	1		Nemertini	1		Chordata	3313
Porifera	6		Mollusca	449		Metazoa	1
Cnidaria	54		Phoronida	1		Tier (animal)	38
Rotifera	2		Nematoda	32		Wirbellose (invertebrates)	7
Acantocephala	1		Tardigrada	1			

Based on 8634 of the 9383 articles in which the name of a taxon was extracted from the title. The time of downloading the files was March 9, 2023

Arthropoda and Chordata comprise more than 80% of the publications. Fifty-eight percent of the taxa are represented by only 1 publication, 93% by less than 10 (Table S3). One percent of the taxa reached more than 60 publications. The honeybee (*Apis mellifera/mellifica*) is the taxon with most publications, followed by locust (*Locusta migratoria*), crayfishes (*Cambarus* spp.), and fruit fly (*Drosophila melanogaster*) (Table S4). A total of 1842 (20%) publications stem from the nine taxa with the most publications.

The article most cited, according to the data analysis of Springer,^3^ is an article on *Drosophila* behavior (Tully and Quinn 1985) with 989 citations. The 50 most cited articles cover 30 taxa: *Drosophila* (*melanogaster*) [3 articles],^4^ (honey) bee [9], nocturnal rodents [5], hymenopteran insects [1], desert ant (*Cataglyphis*) [2], birds [3], fish or Fisch [2], housefly (*Fannia canicularis*) [1], hoverfly (*Syritta pipiens*) [1], animals [2], mouse [1], blue tit (*Parus caeruleus*) [1], blackbird (*Turdus merula*) [1], Weddell seals [1], silk moth (*Bombyx mori*) [1], barn owl (*Tyto alba*) [2], insect [1], red wood ant (*Formica rufa*) [1], gerbil [1], dragonfly [1], fly [1], Elritze [1], invertebrates [1], vertebrates [1], Chiroptera-Rhinolophidae [1], Djungarian hamster (*Phodopus sungorus*) [1], sphinx moth (*Manduca sexta*) [1], blowfly *Calliphora* [1], house mouse [1], bat [1], without taxon name [4]. This list of taxa extracted from the titles of the articles might give the reader a hint of the difficulty of being stringent in naming taxa (see above and footnote 2). More importantly, the large number of taxa in this list underscores the above-mentioned result of the wide variety of taxa represented in the journal. This is also reflected in the themes examined in the top 50 articles that include learning and memory (5 articles), circadian rhythms (8), visual (15) and auditory (5) processing and behavior, orientation (3), locomotion (1), social behavior (3), magneto- (1), mechano- (1), and chemoreception (3), flight behavior (1), metabolism (1), methods (1), and social stress hypothesis (1).^5^

The number of new taxa added to the portfolio was the highest in the 1960/70 s. Three hundred and twenty new taxa were added from 1960 to 1972, a period that included 1029 publications (Table 2). By contrast, in the period from 1994 to 2000 that included 977 publications, only 107 new taxa were added to the list. However, there was not a steady decline in new taxa added because since 2001, the number of new taxa added has again increased.

**Table 2:** New taxa added in the periods covering ~ 1000 publications

Number of publication	1–270	271–1264	1265–2293	2294–3287	3288–4279	4280–5262	5263–6348	6349–7325	7326–8347	8348–9383
Years	1923–1929	1930–1959	1960–1972	1973–1977	1978–1981	1982–1986	1987–1993	1994–2000	2001–2010	2011–2023
Difference	270	994	1029	994	992	983	1086	977	1022	1036
New taxa	169	269	320	197	210	128	122	107	146	193

The variable numbers are a consequence of analyzing the publications as published in full years

The latter result is surprising, on the one hand, since some granting agencies like the National Institutes of Health (NIH) currently concentrate their money on few model systems (see below). On the other hand, the result may be expected because researchers have turned away from studying one question in one species to obtain a more general overview of an issue by studying a broader variety of species. If the numbers extracted from this journal are representative, this might indicate that most granting agencies are still supporting a wide variety of taxa. However, the issue is complex because since 2000 articles from more regions of the world have been published in the journal (we are not aware of a quantitative analysis of the origin of articles), which may have contributed to the rise in the number of new taxa.

The high diversity of taxa found in the publications aligns well the adjective "comparative" in the name of the journal. Comparative in this context specifies the openness of the editors to the inclusion of many taxa that are today not considered as model systems.

What does this historical analysis mean for future research? In our opinion, the results make clear that there are many interesting questions to be tackled and many taxa or "systems" available for answering questions that are relevant now and in future. Therefore, one of the most basic questions for a researcher is to choose the problem, method and organism, she or he wants to examine. Sometimes, the decision is driven by personal motives. Some persons do not want to work with vertebrates; others aren’t attracted by invertebrates. Moreover, some researchers don’t want to use invasive methods, while again others don’t want to go out into the field. However, in each of these areas, much is to be detected, and most, of course, if all different areas are considered. All these motives—and many more that we did not mention here—are also important for the work of neuroethologists. We would like to add one more motive that is especially important for neuroethologists and is based on the belief that there are some questions best examined in specialists, or animals that stand out because of their adaptations. By contrast, neuroethologists working on questions not related to specific adaptations may be subject to considerable competition from researchers working with one of the established model systems. We shall present such an example later: the study of visual motion in flies. While we focus on neuroethology here, the arguments that we develop have, in our opinion, a more general impact and also apply to other disciplines.

We would first like to return to the term "model system". In our view, this term has undergone a change in use in the last forty years. In general, the term means that certain organisms are better suited for answering specific research questions than others. Until the 1980s, the term "better suited" referred to adaptations like those found in snails, bees, locusts, barn owls, bats, etc.. Today, these animals are sometimes referred to as "exotic" animals. Nowadays, the attribute "better suited" reflects some general characteristics that one may find in textbooks: The organism used needs to be easy to rear and to handle, it needs to be inexpensive to keep, available in high quantity, and have a short generation time. Last, but not least, the organism used should be genetically tractable. Based on these criteria, the NIH selected 13 species that it regards as animal model systems for biomedical research (see e.g., Zupanc and Rössler 2022). The NIH, thus, set a focus on the importance of a taxon for research in relation to human health. For neuroscience and ethological questions, well-established model systems from the list of NIH include rhesus monkey (*Macaca mulatta*), rat (*Rattus rattus*), house mouse (*Mus musculus*), zebrafish (*Danio rerio*), fruitfly (*Drosophila melanogaster*), and a nematode (*Caenorhabditis elegans*). Many different questions are examined with these organisms. A look at the selection criteria demonstrates that they reflect features that are often not directly related to a research question. In the following, we shall argue that the research question to be examined should be key when neuroethologists select a species for research. This argument has at least three aspects: first, some animals have developed senses and behavior that are not present in others. Examining the properties of these senses is by itself an important scientific endeavor. Second, animals that stand out because of specific adaptations show better what nature can accomplish in terms of extreme performance, and results obtained from studying such specialists may thus serve better for biomimetic applications than results obtained from studying generalists. Third, similar or different solutions to a problem existing in nature will give us some insights into the solutions found during the evolution of humans. Thus, what we advocate here is an alternative strategy to that developed by the NIH for the selection of a model system. We propose that the study of animals with specific adaptations, specialists, may yield interesting insights that cannot be gained with the current, typically generalist model systems. These insights might be important not only to understand the ability of nature in generating variable solutions but also serve as role models for engineering and other solutions, including biomedical solutions.

In the following, we first will briefly review a selection of past major contributions of work on specialists and their impact on science and society. Finally, we shall exemplify the benefits of working on animals with specific adaptations using mainly three of the systems we have worked on for a long time.

## Past major contributions of work on specialists

Even before the term "neuroethology" was coined, work on specialists provided major new insights into neural processes. Such approaches followed Krogh’s Principle: "For a large number of problems, there will be some animal of choice, or a few such animals, on which it can be most conveniently studied" (Krogh 1929). An eminent example is the work on the giant axon of the squid that led to the quantitative description of the processes underlying the action potential (Hodgkin and Huxley 1952). The giant squid axon, with a diameter of 1 mm, allowed placement of more than one electrode into its lumen, allowed the replacement of ions in the cytoplasm, etc. By contrast, axons with a maximum diameter below 100 μm, as found in mammals, were not a choice at that time. The understanding of the action potential and its propagation had an enormous influence on the understanding of neurological diseases and their treatment, for example amyotrophic lateral sclerosis and multiple sclerosis (Waxman 2002). In a similar way, the high concentration of the nicotinic acetylcholine receptor in the electric eel and the electric ray made it possible to isolate and characterize this protein as the first receptor protein ever (for a review see Changeux 2020). The significance of this finding can be seen in many areas from the design of insecticides, neonicotinoids, to the understanding and treatment of myasthenia gravis (e.g., Mané-Damas et al. 2022). Another success story is the examination of the processes underlying learning, for examples in the snail *Aplysia*, which was chosen for its low numbers of large neurons that were decentrally organized in ganglia (Kandel 2006). These findings paved the way to our understanding of synaptic functioning, molecular pathways, and in the end diseases like schizophrenia and depression. Finally, more current work on the hormone system of prairie and meadow voles has led to the detection of oxytocin and vasopressin as important substances in pair bonding (Froemke and Young 2021). These hormones are also now widely discussed in the context of explaining human behavior. Again, this list could be extended by more success stories, but we stop here. This of course does not mean that we regard other studies as less important or impactful.

## Examples from flies, owls, and weakly electric fish

One may ask whether neuroethologists bother with the "burden" of trying to convince granting agencies to give them money to study their species of choice, when it might be easier to receive support for working with a current model system. We believe that for most neuroethologists, the answer to this question is straightforward: the adaptations of specialists that lead to superior behavior are more attractive than the examination of a similar question in a non-specialist. Moreover, as Walter Heiligenberg pointed out, brains of specialists typically show "a richer complexity in the orchestration of basic designs that they share with simpler organisms" (Heiligenberg 1991). Specialists exhibit enlargements and more ordered brain structures as well as molecular and physiologically efficient processes involved in the processing of specific information. Altogether, these adaptations make specialists very attractive for research. Excellent examples for these claims come, among others, from motion vision in insects, sound localization in owls and object location and communication in weakly electric fish, as we shall detail in the following.

## Motion vision in insects: flies, beetles, etc.

Flies are true masters of the air. They have very short reaction times. It is very hard for us humans to catch flies because they register even slow movements and typically escape quickly before we can strike. Flies are able to land upside down on a ceiling. They chase small objects flying by. They dart back and forth, often returning to the starting point. They patrol along landmarks, e.g., below a lampshade or along a road. They exhibit shadowing, circling, and wobbling behavior. Some fly species are able to hover and to actively fly sideways. Flies typically change flight direction by saccades. These behaviors result by interaction of the flight motor with sensory (feedback) information, importantly from the visual system and are to a large part driven by a neural system that extracts visual motion.

**Fig. 1:**
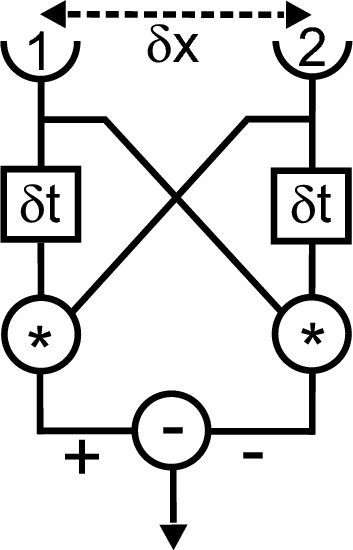
The visual motion detector. '1' and '2' are receptors, δx and δt reflect a spatial shift and time delay, respectively, '*', ' + ' and '−' represent a multiplication, an addition and a subtraction, respectively

**Fig. 2:**
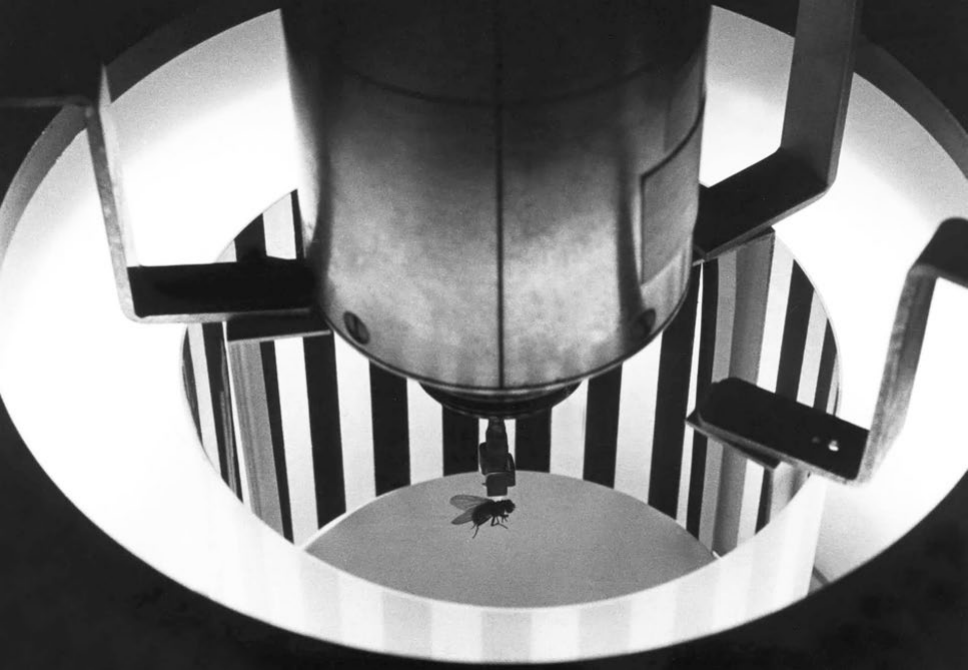
A fly in the torque compensator. This historic picture is published with permission of the Max-Planck Society ( © MPI für Neurobiologie—Robert Schorner/Blachian)

The quantitative study of visual motion in insects started with behavioral experiments on the beetle *Chlorophanus viridis* that led to the extraction of an algorithm that captured the essence of the insect’s behavior induced by visual motion (Hassenstein and Reichardt 1956). This algorithm is known as the Reichardt or Reichardt–Hassenstein movement detector (Reichardt 1987; #49 of most cited articles in the journal^6^). This algorithm is still one of the best models for explaining the extraction of visual motion direction (Fig. 1) (for a recent review see Borst et al. 2020, #1137). After the work with the beetle, Werner Reichardt chose the housefly (*Musca domestica*) to further study visual motion and orientation. The choice was based on the one hand on the virtuosity of fly flight, and on the other hand on reasons of practicability (availability year-round, easy breeding, size, reliable flight behavior), not because the housefly is a specialist for visual motion detection. Experiments were initially carried out with tethered female houseflies. The main reason for choosing females and not both sexes was that female flies were more reliable fliers than male flies. Flies start to fly if the legs loose contact to a rigid object. The trick was to glue a small cardboard triangle on the dorsal thorax of the fly and lift it off the surface. The animal started to fly. The tethered fly was then placed in an instrument, the torque compensator, which allowed the measurement of the yaw torque exerted by the fly—either without a visual surround or while it was stimulated with a visual pattern (Fig. 2). However, the fly remained completely stationary in the compensator. Instead, the angular movement the fly tried to generate was measured, compensated, and used to steer a visual pattern in exactly the same way the fly would have seen the pattern to move would it have been able to turn by itself. A major difference between the tethered flight in the torque compensator and free flight was that the halteres, which act as a kind of gyrosensor, and possibly other sensors, did not provide feedback about the movement of the fly. A big advantage of the experiments in the torque compensator was that the stimulation could be operated in both open and closed loops. In closed loop, the visual stimulation was as it would occur in nature, while in open loop, the visual pattern could be moved independently of the torque generation of the fly. The latter possibility opened the door to carry out a multitude of tests that served to study the feedback circuit underlying visual motion. Overall, this setup allowed quantification of certain behaviors like tracking a wide-field visual pattern or a small object. The systematic examination in the torque compensator of object-induced behavior in front of various types of background constellations resulted in the identification of a position-dependent and a motion-dependent term involved in behavioral control, while other (less systematically driven) reactions could be subsumed in a Gaussian noise term. Similar setups are still widely used in insect research.

The studies of Reichardt’s group served as a prominent example for an engineering and computational approach to understand a biological behavior. One major objection from the neuroethology community was that such an approach does not adequately account for the fact that object-induced behavior is adapted to a wide range of very different behavioral contexts and, for example, male flies show some specializations for chasing small objects because they chase females in the context of mating behavior. By contrast, the approach of Reichardt's group only focused on a rather limited area of the much broader behavioral repertoire. Fly behavior was reduced to a handful of modules, as Marr wrote (1982, p. 32): "Roughly speaking, the fly's visual apparatus controls its flight through a collection of about five independent, rigidly inflexible, very fast responding systems". Marr listed the landing system, the horizontal control system (tracking with a fixation and a dynamic component), and the vertical control system. A change in the picture came through studies of fly free-flight behavior in more natural surroundings, specifically of the chasing behavior of males of the lesser housefly (*Fannia canicularis*) (Land and Collett 1974; #20) and a hoverfly (*Syritta pipiens*) (Collett and Land 1975; #15). These authors demonstrated a much broader spectrum of behaviors, especially in the specialized hoverfly (e.g., shadowing, circling, wobbling (see above)). Even more specialized are marchflies, in which males, but not females have divided compound eyes, with one part directed to the dorso-frontal visual field, which they use to chase females (Zeil 1983a, b; #761, #1018). The fly story is, however, not complete without the bigger blow fly (*Calliphora erythrocephala*) which was used in most electrophysiological studies, because it was much easier to record from *Calliphora* and its relatives than from *Musca* (Hausen 1981; Hengstenberg 1982; #43). Another cornerstone in this research is *Drosophila melanogaster*, one of the current model systems. *Drosophila* offered the possibility to study the influence of mutations on behavior from early on (Heisenberg et al. 1978; #150), but electrophysiology in *Drosophila* then was even harder than in *Musca*, because of its smaller size. This changed dramatically with the advent of new technologies, making *Drosophila* the current standard model of research on fly motion vision (see below).

The experiments carried out with this mix of species allowed many important insights into fly motion vision. More information came from studies of other insects as, for example, dragonflies, locusts, bees and moths. Some important findings are detailed in the seven examples listed in Table 3. We have already mentioned the Reichardt movement detector as derived from initial behavioral experiments (Table 3, #1). This scheme has been basis of many biomimetic approaches of which we just mention one, an algorithm that allows contrast-independent motion detection (Babies et al. 2011). Further behavioral studies demonstrated that the motion input is shaped by active flight strategies in behaviors like cruising, chasing, landing, shadowing, etc. (Collett 1980; #687) (Table 3, #2). One goal thereby is to separate rotational and translational flow (Egelhaaf 2023). The understanding of the neural implementation of the proposed circuit has been a challenge that is still ongoing (see Table 4). However, early on, it became clear that flies have a specific visual pathway for the extraction of motion direction (Table 3, #3). While it had long been known that the pathway includes receptor cells R1-6 at the onset, the finding of a split into an ON and OFF pathway, starting in the lamina (Table 3, #4) was the beginning of the extraordinary fruitful period of research with *Drosophila* mentioned above, that allowed to dissect the pathway, identify its elements, and examine their function. Thus, in the station downstream to the lamina, the medulla, the two pathways again contain several elements with slightly different properties. The neurons T4 (ON pathway) and T5 (OFF pathway) receive inputs from medullary neurons and are the first cells that carry the elementary motion signal, with T4 residing in the medulla and T5 in the lobula. Four subtypes exist for each of the T4 and T5 neurons. Each subtype is sensitive to one of the cardinal directions (Table 3, #5). On a population level, six T4/T5 subtypes were identified. The average tuning of the subtypes shows tuning to diagonal rather than cardinal motion directions (Henning et al. 2022). Both T4 and T5 neurons feed into wide-field lobula plate neurons. The latter generate a multitude of direction selective responses that cover the different flow situations an insect may experience through combinations of excitatory and inhibitory inputs (Table 3, #6). Neurons from the visual pathway feed into several central brain structures and finally into descending neurons, each of which has a specific function in generating behavior (Table 3, #7).

**Table 3:** Important insights from research on insect motion vision in the past

#	Adaptation	Function	References
1	Spatial correlation, input asymmetry and multiplication	Allows the animal to track the direction of motion of a visual pattern	Hassenstein and Reichardt (1956); Yang and Clandinin (2018); Borst and Groschner (2023)
2	Active flight strategies shape retinal motion	Allow to separate, for example, rotational and translational flow	Collett (1980); Cellini and Mongeau (2020); Fenk et al. (2021); Egelhaaf (2023)
3	Specific visual pathway for the extraction of motion direction	Computation of motion direction independent of other visual attributes	Heisenberg and Buchner (1977; #54); Silies et al. (2013)
4	Separate ON and OFF pathways	Separate computation of increments and decrements of light	Riehle and Franceschini (1984); Joesch et al. (2013); Behnia et al. (2014); Leonhardt et al. (2016)
5	Four subtypes in each pathway, six on the population level	Each neuron represents of one of the cardinal directions; on the population level diagonal directions are represented	Yang and Clandinin (2018); Borst et al. (2020); Henning et al. (2022); Shinomiya et al. (2022)
6	Wide-field neurons in the lobula plate	Integration of many elementary motion detectors to generate responses specific for different flow situations	Hausen (1981); Karmeier et al. (2005); Mauss and Borst (2020); Krapp and Hengstenberg (1996)
7	Several central brain structures and specific descending neurons	Independent computation of different behaviors: navigation, feature extraction, context dependence, reflexes	Ache et al. (2019); Cheong et al. (2020); Namiki et al. (2022); Ryu et al. (2022); Currier et al. (2023)

**Table 4:** Interesting current and future questions of fly visual behavior

#	Topic
1	What is the exact realization of the elementary motion detector? Is there only one realization of the motion detector in insects or more?
2	Synaptic, molecular and biophysical mechanisms underlying motion detection
3	Modulatory influences on motion vision
4	Context-dependent and cognitive influences on motion vision
5	Role of motor feedback in different motion-based behaviors
6	Male-specific chasing behavior and the underlying visual and motor pathway
7	Species-specific adaptations to use optic flow for migration, navigation, collision-free traversal of dense, cluttered environments

In the context of our arguments, the studies of visual motion in flies may serve as an example for a research direction in which, as methodology improved over time, work on a non-specialist model system, *Drosophila*, outcompeted not only the work on the other non-specialists that are not regarded as model systems, *Musca* and *Calliphora*, but also more specialist flies like hoverflies and marchflies. There is no doubt that research in *Drosophila* will also dominate motion-based fly research in future and that these future studies might eventually solve the question of "How does the fly compute visual motion?". However, important details in this direction are still missing and/or are currently controversial. In Table 4, we list 7 topics that are interesting in our view and deserve more examination in future. One of these questions relates to the exact structure of the motion detector, specifically on how preferred direction amplification and null direction suppression are implemented (Table 4, #1). A side question that goes beyond research in *Drosophila* and even flies is whether there is only one realization of the motion detector in insects or more. While much is known about the computation at the cellular level, synaptic, molecular and biophysical mechanisms need more examination (Table 4, #2). Other important aspects that have already yielded some results, but need more in-depth examination, are the influence of pharmacological modulation on the detector and its elements (Table 4, #3). It has already been shown, for example, that both serotonin and octopamine modulate visual processing (Cheng and Frye 2020; #4257). The expectation is that many more modulatory influences will be unraveled in future. Likewise, context-dependent studies have already yielded interesting results in relation to motion vision, among others for looming stimuli (Oram and Card 2022). This field is only in its infancy. While, cognitive and contextual influences on fly behavior were long considered to be absent, we expect future research to unravel more capabilities of insects. We note that researchers need to be careful not to over-interpret complex behavior in terms of human behavior (Table 4, #4). Insect behavior also includes feedback from the motor to the sensory side by an efference copy (Kim et al. 2015) (Table 4, #5). There are also questions that cannot be investigated in *Drosophila*. One of these questions is male chasing, which may best be investigated in specialists like hoverflies or marchflies. Such examinations should not only involve the behavior (Thyselius et al. 2023), but also the visual pathway underlying chasing (Nicholas et al. 2020; #6684) (Table 4, #6). It is a challenge for those who like to study such adaptations in future to bring the species with the specializations back to neuroethology. Other issues relate to adaptations for the use of optic flow in migration, and navigation via collision-free cruising in cluttered environments (Table 4, #7).

## Sound localization in owls

**Fig. 3:**
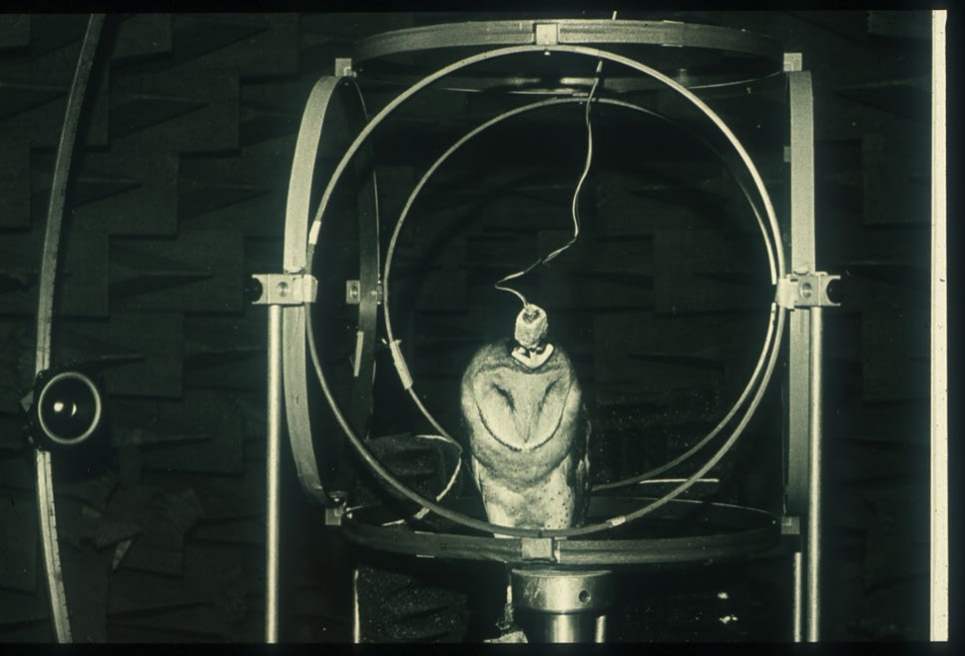
Early sound localization setup with barn owl. The big Helmholtz coils and a small head coil allow measurement of head movements in azimuth and elevation. The loudspeaker, movable along a hoop, on the middle left, was used to acoustically stimulate the owl

Ever since Roger Payne published his seminal article on barn owl sound localization (Payne 1971), this species has been one of the most successful model systems in neuroethology. Barn owls are nocturnal hunters, with many mechanical, auditory, and visual adaptations that make them very successful predators. Most conspicuous is the ruff of the owl. The German (Schleiereule = “ruff owl”), but not the English name (barn owl), includes this characteristic (Table 5, #1) (Fig. 3). The physiological function of the ruff is to increase auditory sensitivity. Hidden by the ruff feathers are large vertically asymmetric ear openings (Table 5, #2). This vertical asymmetry allows the owl to use a binaural parameter, interaural level difference, for vertical localization and, thus, improves spatial resolution in this direction (Moiseff 1989a + b; #788, #435). No less conspicuous than the ruff are the large head movements of the owls. Vertebrae and muscles allow rotations of more than 270° (Boumans et al. 2015). This ability compensates for the small range of eye movements. The exquisite auditory sensitivity would not help the owl if it would make noise with its wings during hunting flights that constitute about 50% of hunts. This is one possible reason why the owl has specific adaptations that suppress noise generation during flight (Table 5, #3). Further specializations are that the barn owl does not have an epiphysis, and that it has branched fatty acids in its uropygial gland that show promising characteristics for use as industrial lubricants, if cultivated in genetically engineered plants (Biester et al. 2012). However, especially the results related to its sound localization capabilities have had impacts beyond the neuroethological community.

**Table 5:** List of 7 adaptations of owls for specialized sound localization

#	Adaptation	Function	References
1	Ruff	Enhances auditory sensitivity	Hausmann et al. (2009)
2	Vertical ear asymmetry	Allows high spatial resolution in vertical space coordinate	Payne (1971); Moiseff (1989a, b)
3	Silent flight	Allows the owl to sense faint sounds, camouflage	Wagner et al. (2017); Clark et al. (2020)
4	Enlarged auditory brain structures	Leads to more efficient processing	Wagner et al. (1987); Carr and Konishi (1990)
5	Auditory fovea, increased representation of frequencies between 5 and 10 kHz	Extraction of interaural time difference and interaural level difference in the same frequency band	Koeppl et al. (1993); Koeppl (1997)
6	Adaptation of auditory processing to input specifics	Improves efficient processing	Cazettes et al. (2014); Shadron and Peña (2023)
7	Bimodality in almost all neurons in optic tectum	Integrates visual and auditory information	Knudsen (1982)

**Fig. 4:**
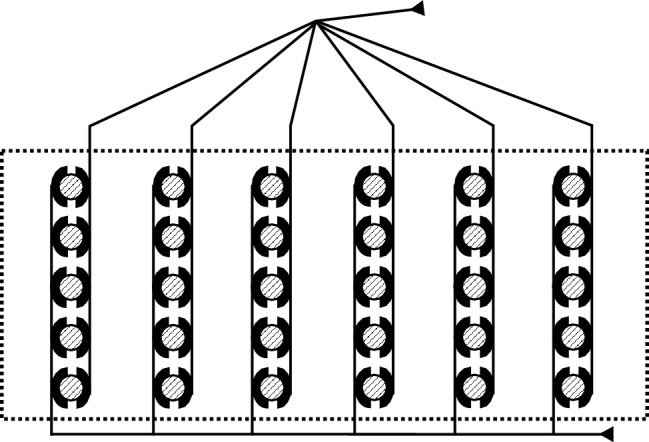
The Jeffress model for the extraction of interaural time difference. The lines represent axons from cells located in nucleus magnocellularis that function as delay lines with arrowheads indicating the direction of the flow of information. The round elements represent somata in nucleus laminaris (delineated by the dotted line) that function as coincidence detectors

The evolutionary pressure acting on this nocturnal hunter has resulted in behavior and neural circuits that are among the most effective in nature (Konishi 1993; #5107). This is mediated by larger brain structures for processing of auditory information than in non-specialized organisms like the pigeon or the chicken. These nuclei are also geometrically more structured and thus easier to study (Table 5, #4). For example, owls possess an auditory fovea with an enhanced resolution for frequencies between 5 and 10 kHz (Table 5, #5). This frequency range is higher than is typically found in birds, but interaural amplitudes are more salient at these frequencies than in lower frequency ranges. By contrast, 5 to 10 kHz is out of the range in which mammals typically can extract interaural time difference, a second important binaural parameter that mainly varies with azimuth. The ultimate function of these adaptations is that they endow the owl with the ability to measure binaural sound localization parameters in two dimensions in the same frequency range with high resolution (Knudsen and Konishi 1979, #27; Knudsen et al. 1979, #29; Moiseff 1989b, #435). Furthermore, the circuit to extract interaural time difference, the cue of the acoustic signal that many animals, including the barn owl, use for azimuthal sound localization, reflects a very efficient solution (Fig. 4). This circuit implements a process that contains delay lines and coincidence detection, very close to a model proposed by Jeffress (1948). Formally, the computation is similar to correlation. Spike arrival times at the soma, depending on frequency-specific external and internal delays, are evaluated to create an ambiguous signal for a location in space.

An unambiguous signal for a location would result from broad-band correlation, and indeed from a theoretical point-of-view, it would be more effective if the correlation would occur across the whole range of available frequencies, but the peripheral auditory system does not provide this information. The barn owl brain deals with this problem by neural computation that leads to the representation of broadband, across-frequency information in several more steps in the so-called space-specific neurons (Knudsen et al. 1977; Takahashi and Konishi 1986; Wagner et al. 1987). These neurons represent a location in space, exactly what the owl needs to catch a mouse. Different neurons are tuned to different locations, and many neurons together represent the map of auditory space (Knudsen and Konishi 1978). The computations leading to the space-specific neurons include, again, a multiplication. This multiplication is very close to a perfect mathematical multiplication, a very rare case of an almost ideal biological computation (Peña and Konishi 2001). While we argued that broad-band correlation would be most effective from the start, the initial frequency-specific computation also has its advantages. It allows different across-frequency integration in the two major auditory pathways, the tectofugal and the thalamofugal pathways. This may be seen as an example of what Heiligenberg (1991) meant by "richer orchestration" (see also Vonderschen and Wagner 2014). Whereas the tectofugal pathway yields more precise spatial information, and is thus most important for the localization process, the thalamofugal pathway integrates information from other sources, including non-auditory and cognitive information. There is also feedback from the thalamofugal to the tectofugal pathway.

As said before, the tectofugal pathway represents locations very precisely. It does this even more specifically than explained so far. The space-specific neurons are adapted such that they reflect the reliability of the incoming frequency information (Table 5, #6). High-frequency information is more reliable in frontal space than in lateral space, and the bandpass neurons representing frontal space are tuned to higher frequencies than those representing lateral space. This very efficient auditory pathway of the barn owl has been an inspiration for the construction of sound localization devices in a biomimetic sense (Lazarro and Mead 1989; Das et al. 2019). Auditory information is combined with visual information in the optic tectum. Almost all tectal neurons are bimodal and carry both visual and auditory information (Table 5, #7). Electrical stimulation of tectal neurons elicits head turns that are directed to the position in space that corresponds to the external location represented in the tectal map at the location stimulated (duLac and Knudsen 1990).

Being so successful in the past, one may ask what novel insights can be found in future? This question was already brought up by our mentor, Mark Konishi, when two of us were postdocs in his lab between 1984 and 1988. Mark expressed the opinion that "The gold is off the owl". At that time, mainly the map of auditory space was known, but not the computational processes leading to the map. The last 30+ years have shown that much gold was still to be discovered. This leads us to ask: What is missing as of today? One may further ask whether, in future, research on sound localization in mice might make research on owls superfluous. Thus, the next question is, what is largely specific for the owl?

We list seven examples below. Roulin (2020) covered mainly evolutionary and ecological aspects of barn owl biology, while neuroethologists so far have been mainly interested in behavior and neural processing. These different themes overlap, but the scientific integration is only in its infancy (Table 6, #1). Along this line, many interesting aspects could be followed up like, for example, whether the main function of the silent flight is self-masking (i.e., the owl improves its own hearing by reducing self-produced noise) or stealth (prey cannot hear the predator, because it does not produce sound) (Clark et al. 2020)? Aspects of sensory ecology have been examined by the Peña group (Cazettes et al. 2014; Shadron and Peña 2023) and should be extended. We need not only know how the brain represents the environment, but also how this information is back-projected from the brain to the environment for successful survival. For example, the acoustic environment is most completely represented by head-related transfer functions (HRTF). Inverse modeling may help to find out which parts of the information in the HRTFs are used in behavior and how this information is represented in neurons (as examined in a pilot study by Schillberg et al. 2020; #8918). In a broader sense, the question arises as to how well single cells represent input specifics. There is already some evidence that a population vector readout of the neuronal responses in the space map can mediate statistical inference in sound-localizing behavior of barn owls (Ferger et al. 2021).

**Table 6:** Interesting current and future questions of owl research

#	Topic
1	Combine neuroethological approaches with those of ecology and evolution: e.g., function of silent flight; inverse problem; adaption of neural processing to the incoming signal (ultimate and proximate relation)
2	Manipulation of map of auditory space or almost perfect multiplication by optogenetics or CRISPR/Cas
3	Non-hypothesis driven approach by genomics, transcriptomics, metabolomics, and connectomics to examine molecular realization of short action potential
4	Genetic basis of enlarged auditory nuclei and of ear asymmetry
5	Cortex-like organization of sensory part of forebrain and computations like those underlying stereo vision
6	Limits of cognitive behavior
7	Neck mobility

A further challenge is to develop genetic tools (optogenetics, CRISPR/Cas) for owls to be able to dissect the sound localization circuit (Table 6, #2). This should be especially useful for neural computations in which owls show specializations. One prominent example is the map of auditory space in the external nucleus of the inferior colliculus already mentioned. Another, most likely no less interesting example, is the combination of information derived from interaural time and level differences accompanied by broadening of frequency tuning in the lateral shell of the inferior colliculus (Mazer 1998). Likewise, the resolution of 10 μs, as it occurs in the processing of interaural time difference, needs a very short and fast rising action potential. Standard voltage sensitive sodium and potassium channels seem too slow.

The comparison of genomics data of the owl with those of non-specialists may yield insight into possible specializations of owl ion channels, synaptic components, and in the end the high physiological efficiency of the neural algorithms (Table 6, #3). Such research might also produce findings related to the basis of the enlarged auditory structures in the owl. Furthermore, a genetic study of the ear asymmetry, which varies even within the owl clade, and its comparison to other asymmetries in the body like the heart, liver, etc. may yield interesting insights into the molecular basis of asymmetry (Table 6, #4). Not only are auditory structures enlarged, but also the sensory forebrain part is more complex in owls than in pigeons (Stacho et al. 2020). This is based on a large visual Wulst that harbors many neurons involved in the computation of depth by stereo. Studying these specializations would also most likely lead to new insights of neural processing in general, specifically of mechanisms underlying stereo vision, audition, and maybe olfactory processing (Table 6, #5).

Birds exhibit surprising cognitive capabilities. However, the limits of cognitive capabilities of birds, including owls are not yet clear. To this end, behavioral experiments probing working memory, theory of mind, visual search, motivational circuits (owls need to care about when to spend energy in hunting), etc. should be carried out (Table 6, #6). Some information is already available on the representation of space in the hippocampus, in other words beyond the space map in the midbrain (Agarwal et al. 2023).

An anatomical specialization we like to remember is the highly flexible neck (Table 5, #3), which is a challenge not only for the muscular arrangement but also for the paths of blood vessels and nerves. Little has been done in this direction so far (de Kok-Mercado et al. 2013), and further studies might reveal clever biological solutions to prevent squeezing or crushing of blood vessels and strain on nerves (Table 6, #7). There are many more areas that could be scrutinized and examined before we will understand how all neural and non-neural specializations have evolved in concert to allow the owl to be the efficient predator it is today.

## Electroreception in weakly electric fish

It was hard to miss the presence of strongly electric pulses among fishes, even before electricity was understood (Kellaway 1946). By contrast, the existence of weak electric signals and their use in communication and object detection was only discovered in the 1950s (Lissmann 1951; see also review by Pitchers et al. 2016). Electric fish live in turbid water and are active mainly at night. Therefore, they cannot use vision for communication and object detection. The electrosensory system has become a model for spatial senses, and more importantly, one of the best understood models for following the connection of relevant sensory coding to motor decisions (Heiligenberg 1973, #136; Bastian 1976, #374; Rasnow 1996, #191). The generation of electric signals is based on the transformation of myocytes or axonal terminals to electrocytes (Table 7, #1). Electrocytes may be arranged in parallel to sum up their currents or in series to sum up their voltages, following Kirchhoff’s rules.

**Table 7:** Seven important past findings in research on weakly electric fish

#	Adaptation	Function	References
1	Transformation of myocytes to electrocytes	Generation of electric potential without action potential	Darwin (1872)
2	Pulse—wave fishes	Two different signal types that serve the same function evolved in parallel	Lissmann (1958)
3	Circuit of electrosensory lateral line, enlargement of brain structures involved in processing of electric signal, different receptor types and 3 parallel maps	Computational principles underlying contrast coding, high-resolution extraction and processing of different aspects of the electric signal	Hopkins (1976; #69); Heiligenberg and Dye (1982; #288); Shumway 1989; Clarke et al. (2015)
4	Temporal resolution below 1 μs	Processing of communication signal	Carr et al. (1986)
5	Coarse coding and "neural democracy" in generation of jamming avoidance	Increase resolution, generate most likely signal to reduce beating	Heiligenberg et al. (1978)
6	Hormonal influences on electrocytes	Male courtship signals contain information about fitness	Hopkins (1972); Zakon et al. (2008); Silva Barbato et al. (2020)
7	Descending connections, specifically corollary discharge and efference copy	Discrimination between own signal output and contaminated input	Bell and v.d. Emde (1995; #3158)

Studies of this simple motor system have been models for the genetics of voltage-gated channels (Zakon et al. 2006) and for the roles of hormones in the control of electric signals (Hopkins 1972). Other key features of the electrosensory system include the parallel evolution of active weak electric senses in South American Gymnotiformes and African Mormyriformes (Bullock et al. 1983; Kawasaki 2009).

**Fig. 5:**
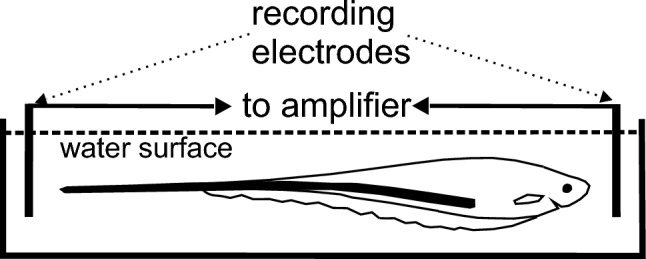
Sketch of the weakly electric, wave-type fish *Eigenmannia* in a recording setup

The attractiveness of the communication behavior of weakly electric fish for neuroethology is that a complete behavioral circuit can be studied in a controlled experimental setup (Fig. 5). Even in restrained conditions, the fish actively generates an electric field, senses distortions of this field, and generates motor output accordingly. A further great advantage is that fish tolerate curarization. In this situation, the natural electric field can be replaced by an artificial electric field that allows studying important computational and behavioral parameters under open-loop conditions (Heiligenberg 1991).

Not only can the complete behavioral circuit be studied in weakly electric fish, but the studies of this circuit also lends itself to exploration at various levels. The integration of behavioral, anatomical, physiological, and molecular techniques have led to a deep understanding of electro-communication and object detection in weakly electric fish.

**Fig. 6:**
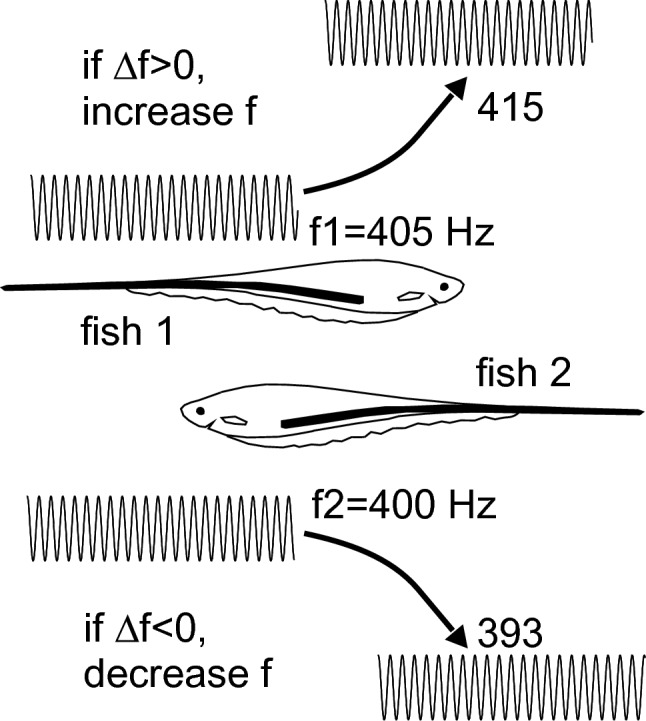
The jamming avoidance response. The original frequencies of the wave-type electric organ discharge of two fish are 405 Hz (fish 1) and 400 Hz (fish 2), respectively. When the fish come close together, fish 1 increases its frequency to 415 Hz, while fish 2 decreases its frequency to 393 Hz to avoid jamming

One of the major early insights was that different species of electric fish use two different types of signals: pulses emitted at variable intervals and continuously produced sinusoidal waves of a given frequency. This is a nice example of two different signal types serving similar functions (Table 7, #2). One of the best examined behaviors of electric fish is the jamming avoidance that is observed in wave species. If two wave-type fish with similar frequency are close to each other, beat frequencies arise that make it difficult for the fish to use the electric signal for electrolocation. The fish avoid this situation actively by shifting their frequencies apart, a behavior termed jamming avoidance (Watanabe and Takeda 1963) (Fig. 6). Jamming avoidance needs both a precise temporal and a reliable amplitude information, provided by specialized receptors. Wave-type fish have P- and T-type receptors, while pulse-type fish have knollenorgans, mormyromasts and ampullary receptors (Table 7, #3). Each of these types is specialized for processing a certain aspect of the signal. The ampullary receptors measure the electric fields generated by other animals. The A sensory cells in the mormyromasts and the P-units well represent stimulus amplitude. The knollenorgans, the B sensory cells in the mormyromasts, and the T-units convey high temporal resolution. The temporal resolution of weakly electric fish is below 1 μs (Carr et al. 1986) (Table 7, #4), which is exceptional.

Brain structures involved in the processing of electric signals are enlarged and constructed for extraction of behaviorally relevant stimulus features (Krahe and Maler 2014). Maps and columns of the electrosensory lateral line lobe provide parallel information streams tied to different behavioral contexts (Table 7, #3). Each map seems to be adapted to detect objects of a specific size and speed (Shumway 1989). This adaptation is one means of increasing signal bandwidth. Another interesting aspect of the neural processing is what Heiligenberg et al. (1978; #680) called "neuronal democracy" in deciding behavior (Table 7, #5). This represents one of the first well described examples of population coding, and serves to eliminate ambiguities in measuring the jamming avoidance response. The hormonal influences, specifically on the male courtship signal in pulse fishes, allows the female, among others, to detect aspects of male fitness (Table 7, #6). Descending connections also play an important role in the behavior of weakly electric fish (Table 7, #7). In mormyrids, the self-generated stimuli are canceled either by an exact efference copy or by a more general corollary discharge, thus allowing the fish to discriminate between its own signal output and the possibly distorted and contaminated version of the self-produced signal at the input (Fukutomi and Carlson 2020).

Electric fish are attractive not only to neuroethologists, but also to molecular and cellular biologists. The latter are typically more competent in including genomics in their research but are not necessarily interested in neuroethology. An integration of the more behaviorally oriented neuroethological and the more molecular oriented biological research should open up new avenues for research. We have already mentioned the exceptional temporal resolution of weakly electric fish. The molecular basis of this resolution could possibly profit from a comparison with other high-resolution temporal systems like those found in bats and owls (Table 8, #1). Further powerful insights have emerged from the study of specialized proteins of the electrocytes, not only voltage-gated sodium channels, but also other channels, electrogenic and propagation-related proteins that are the basis of the generation of the electric organ discharge (EOD), and, thus, the behavioral capability of the fish (Table 8, #2) (see also Pitchers et al. 2016). Electric fish are an outstanding model for evaluation of sex-specific hormonal influences on the EOD (Zakon et al. 2008; Silva Barbato et al. 2020) (Table 7, #6). As of today, most studies have been conducted on the behavioral and physiological levels. The inclusion of genomics, transcriptomics, metabolomics, and connectomics in this research would lift this research to a new level (Table 8, #3), and might create crosslinks to other systems like birdsong. Electric fish are, furthermore, a model for neurogenesis and neuronal regeneration in the adult fish brain (Zupanc 2006; #163) (Table 8, #4). These cellular advances do not mean that physiological research should be discarded. By contrast, using optogenetics in physiological and behavioral studies of weakly electric fish (Table 8, #5) should allow additional manipulations to dissect the neural circuits as it does in other systems, including zebrafish (for a review see Friedrich et al. 2010). Neuroethological research should not only include more molecular techniques, it should also make use of advanced computational approaches to exploit the possibility of social interactions of fish with robots or the use of supervised learning models (Worm et al. 2021; Pedraja et al. 2021) (Table 8, #6). Moreover, computational modeling of the behavior of weakly electric fish should continue to provide a deeper understanding of behavior (Heiligenberg 1991) (Table 8, #6). This would also help to design further bioinspired agents (von der Emde 2007; Neveln et al. 2013). A large, but rewarding challenge also lies in new techniques to study behavior and neural processing of freely moving fish, either in the laboratory (Fotowat et al. 2019) or in the wild (Henninger et al. 2020) (Table 8, #7), thus overcoming the constraints of restrained conditions.

**Table 8:** Interesting current and future questions of research on weakly electric fish

#	Topic
1	Molecular basis of exceptional temporal processing
2	Function of electrocyte proteins, Na^+^-channels and beyond
3	Use of genomics, transcriptomics, metabolomics, and connectomics to study genetic influences on electric signal and fitness
4	Neurogenesis and neuronal regeneration in the adult fish brain
5	Manipulation of neural circuits using optogenetics
6	Study of behavior through interaction of fish with robot or the use of supervised learning models; computational modeling of behavioral and neural circuit and design of agents
7	Study of behavior in the natural habitat

## Concluding remarks

We have selected the three examples for detailed presentations not only because we have worked with these systems, but also because they represent, in our opinion, different prospects for future neuroethological research:

1. The very successful research in *Drosophila* dominates research on motion vision at the moment. *Drosophila* is one of the current model systems, but this species is not a specialist for motion vision. There are many species with specific adaptations and in some, like bees, dragonflies and hawkmoths, research is thriving. It is promising that also research on specialized fly species continues (Nicholas et al. 2020; Thyselius et al. 2023). The hope is that in future more research with specialist flies and even other insect taxa will be resumed or newly started.
2. Sound localization studies in owls are in competition with similar studies in both avian and mammalian non-specialists. A fascinating result of these comparisons is that the neural algorithm of computing the location of a sound source in barn owls differs from the mammalian algorithm (e.g., see Table 5, #2, 4, 7). Owl research may have been negatively affected by the claim that it does not help to understand human hearing. On the other hand, owl specializations for localization have made this research attractive for biomimetic applications (Das et al. 2019). The hope is that the elegant solutions observed in the barn owl will attract researchers in future.
3. Research on electric fish does not have the same level of competition as the research in the barn owl, because humans do not have a corresponding electric sense. However, by occupying its own ecological niche, it faces the same threats in regards to citations and impact as barn owl research. Even electric fish research may have direct impacts on clinical research, for example through analyses of the molecular mechanisms leading to the conversion of a myocyte to an electrocyte (Zakon et al. 2006).

In this article, we have expressed our hope that future neuroethologists will find the amazing solutions developed by animals in the course of evolution attractive enough to continue working with non-standard species. We agree with Manger et al. (2008) that "the study of ‘nontraditional’ species has yielded novel insights into the functional organization of the brain that would not have resulted from studying only lab species." It will also do so in future.

### Supplementary Information

Below is the link to the electronic supplementary material.

Supplementary file1 (DOCX 12 KB)

Supplementary file2 (DOCX 12 KB)

Supplementary file3 (DOCX 14 KB)

Supplementary file4 (DOCX 13 KB)

Supplementary file5 (DOCX 14 KB)

## Footnotes

1 A similar contribution appears in this issue from the perspective of a young investigator (Beetz).

2 Note that the term taxon/taxa is used somewhat loosely here, because many different names were used for one taxon in the course of time in the journal. Also, the level of using the name of a taxon was not consistent—at least in the titles of the articles that were available to us. For example, some authors used *Rana temporaria*, others *Rana*, others frog or Frosch, … In this case we subsumed all uses under "*Rana* spp. (frogs)". We proceeded in a similar way in other cases.

3 Date of file: 2023-05-15, provided by Gilliard Lach of Springer. Note that the numbers provided by Springer typically deviate from the numbers found in other data bases.

4 Note that the taxonomic notation in the titles of the articles was not consistent. We have not resolved this mixture of use of names. The round brackets behind a taxon name mean that sometimes only the first name was used and sometimes the complete name. The squared brackets refer to the number of articles in the top 50 cited for a given taxon. Note that the sum is higher than 50, because in some titles more than one taxon appeared.

5 Note that the sum is 51, because one article mentioned two research directions.

6 For articles we cite that were published in the Journal of Comparative Physiology we shall use the abbreviation #xx to indicate the rank within the most cited articles in the journal behind the date of publication.

## References

[CR1] Ache JM, Namiki S, Lee A, Branson K, Card GM (2019). State-dependent decoupling of sensory and motor circuits underlies behavioral flexibility in *Drosophila*. Nat Neurosci.

[CR2] Agarwal A, Sarel A, Derdikman D, Ulanovsky N, Gutfreund Y (2023). Spatial coding in the hippocampus and hyperpallium of flying owls. Proc Natl Acad Sci USA.

[CR3] Babies B, Lindemann JP, Egelhaaf M, Möller R (2011). Contrast-independent biologically inspired motion detection. Sensors.

[CR4] Bastian J (1976). Frequency response characteristics of electroreceptors in weakly electric fish (Gymnotoidei) with a pulse discharge. J Comp Physiol.

[CR5] Behnia R, Clark DA, Carter AG, Clandinin TR, Desplan C (2014). Processing properties of ON and OFF pathways for *Drosophila* motion detection. Nature.

[CR6] Bell C, von der Emde G (1995). Electric organ corollary discharge pathways in mormyrid fish. J Comp Physiol A.

[CR7] Biester EM, Hellenbrand J, Gruber J, Hamberg M, Frentzen M (2012). Identification of avian wax synthases. BMC Biochem.

[CR8] Borst A, Groschner LN (2023). Spatial correlation, input asymmetry and multiplication as fundamental characteristic as determined from behavior. How flies see motion. Ann Rev Neurosci.

[CR9] Borst A, Haag J, Mauss AS (2020). How fly neurons compute the direction of visual motion. J Comp Physiol A.

[CR10] Boumans M, Krings M, Wagner H (2015). Muscular arrangement and muscle attachment sites in the cervical region of the American barn owl (*Tyto furcata pratincola*). PLoS ONE.

[CR11] Bullock TH, Bodznick DA, Northcutt RG (1983). The phylogenetic distribution of electroreception: Evidence for convergent evolution of a primitive vertebrate sense modality. Brain Res Rev.

[CR12] Carr CE, Konishi M (1990). A circuit for detection of interaural time differences in the brainstem of the barn owl. J Neurosci.

[CR13] Carr CE, Heiligenberg W, Rose G (1986). The detection of small temporal disparities in the weakly electric fish *Eigenmannia*. J Neurosci.

[CR14] Cazettes F, Fischer BJ, Pena JL (2014). Spatial cue reliability drives frequency tuning in the barn owl's midbrain. Elife.

[CR15] Cellini B, Mongeau JM (2020). Active vision shapes and coordinates flight motor responses in flies. Proc Nat Acad Sci USA.

[CR16] Changeux JP (2020). Discovery of the first neurotransmitter receptor: the acetylcholine nicotinic receptor. Biomolecules.

[CR17] Cheng KY, Frye MA (2020). Neuromodulation of insect motion vision. J Comp Physiol A.

[CR18] Cheong HS, Siwanowicz I, Card GM (2020). Multi-regional circuits underlying visually guided decision-making in *Drosophila*. Curr Opin Neurobiol.

[CR19] Clark CJ, LePiane K, Liu L (2020). Evolutionary and ecological correlates of quiet flight in nightbirds, hawks, falcons, and owls. Integr Comp Biol.

[CR20] Clarke SE, Longtin A, Maler L (2015). Contrast coding in the electrosensory system: parallels with visual computation. Nat Rev Neurosci.

[CR21] Collett TS (1980). Some operating rules for the optomotor system of a hoverfly during voluntary flight. J Comp Physiol.

[CR22] Collett TS, Land MF (1975). Visual control of flight behaviour in the hoverfly (*Syritta pipiens L.*). J Comp Physiol.

[CR23] Currier TA, Pang MM, Clandinin TR (2023). Visual processing in the fly, from photoreceptors to behavior. Genetics.

[CR24] Darwin (1872) The origin of species by means of natural selection, 6th edn. Cambridge University Press, Cambridge, UK. 10.1017/CBO9780511694295

[CR25] Das S, Dodda A, Das S (2019). A biomimetic 2D transistor for audiomorphic computing. Nat Commun.

[CR26] De Kok-Mercado F, Habib M, Phelps T, Gregg L, Gailloud P (2013). Adaptations of the owl’s cervikal & cephalic arteries in relation to extreme neck rotation. Science.

[CR27] du Lac S, Knudsen EI (1990). Neural maps of head movement vector and speed in the optic tectum of the barn owl. J Neurophysiol.

[CR28] Egelhaaf M (2023). Optic flow based spatial vision in insects. J Comp Physiol A.

[CR29] Fenk LM, Kim AJ, Maimon G (2021). Suppression of motion vision during course-changing, but not course-stabilizing, navigational turns. Curr Biol.

[CR30] Ferger R, Shadron K, Fischer BJ, Peña JL (2021). Barn owl's auditory space map activity matching conditions for a population vector readout to drive adaptive sound-localizing behavior. J Neurosci.

[CR31] Fotowat H, Lee C, Jaeyoon Jun C, Maler L (2019). Neural activity in a hippocampus-like region of the teleost pallium is associated with active sensing and navigation. Elife.

[CR32] Friedrich RW, Jacobson GA, Zhu P (2010). Circuit neuroscience in zebrafish. Curr Biol.

[CR33] Froemke RC, Young LJ (2021). Oxytocin, neural plasticity, and social behavior. Annu Rev Neurosci.

[CR34] Fukutomi M, Carlson BA (2020). A history of corollary discharge: contributions of mormyrid weakly electric fish. Front Integr Neurosci.

[CR35] Hassenstein B, Reichardt W (1956). Systemtheoretische Analyse der Zeit-, Reihenfolgen- und Vorzeichenauswertung bei der Bewegungsperzeption des Rüsselkäfers Chlorophanus. Z Naturforschung B.

[CR36] Hausen K (1981). Monocular and binocular computation of motion in the lobula plate of the fly. Verh Dtsch Zool Ges.

[CR37] Hausmann L, von Campenhausen M, Endler F, Singheiser M, Wagner H (2009). Improvements of sound-localization capabilities by the facial ruff of the barn owl (*Tyto alba*) as demonstrated by virtual ruff removal. PLoS ONE.

[CR38] Heiligenberg W (1973). Electrolocation of objects in the electric fish *Eigenmannia* (Rhamphichthyidae, Gymnotoidei). J Comp Physiol.

[CR39] Heiligenberg W (1991). Neural nets in electric fish.

[CR40] Heiligenberg W, Dye J (1982). Labelling of electroreceptive afferents in a gymnotoid fish by intracellular injection of HRP: the mystery of multiple maps. J Comp Physiol.

[CR41] Heiligenberg W, Baker C, Matsubara J (1978). The jamming avoidance response in *Eigenmannia* revisited: the structure of a neuronal democracy. J Comp Physiol.

[CR42] Heisenberg M, Buchner E (1977). The role of retinula cell types in visual behavior of *Drosophila melanogaster*. J Comp Physiol.

[CR43] Heisenberg M, Wonneberger R, Wolf R (1978). Optomotor-blind^H31^—a *Drosophila* mutant of the lobula plate giant neurons. J Comp Physiol.

[CR44] Hengstenberg R (1982). Common visual response properties of giant vertical cells in the lobula plate of the blowfly *Calliphora*. J Comp Physiol.

[CR45] Henning M, Ramos-Traslosheros G, Gür B, Silies M (2022). Populations of local direction-selective cells encode global motion patterns generated by self-motion. Sci Adv.

[CR46] Henninger J, Krahe R, Sinz F, Benda J (2020). Tracking activity patterns of a multispecies community of gymnotiform weakly electric fish in their neotropical habitat without tagging. J Exp Biol.

[CR47] Hodgkin AL, Huxley AF (1952). A quantitative description of membrane current and its application to conduction and excitation in nerve. J Physiol.

[CR48] Hopkins CD (1972). Sex differences in electric signaling in an electric fish. Science.

[CR49] Hopkins CD (1976). Stimulus filtering and electroreception: Tuberous electroreceptors in three species of gymnotoid fish. J Comp Physiol.

[CR50] Jeffress LA (1948). A place theory of sound localization. J Comp Physiol Psychol.

[CR51] Joesch M, Weber F, Eichner H, Borst A (2013). Functional specialization of parallel motion detection circuits in the fly. J Neurosci.

[CR52] Kandel E (2006). In search of memory.

[CR53] Karmeier K, Krapp HG, Egelhaaf M (2005). Population coding of self-motion: applying Bayesian analysis to a population of visual interneurons in the fly. J Neurophysiol.

[CR54] Kawasaki M (2009). Evolution of time-coding systems in weakly electric fishes. Zool Sci.

[CR55] Kellaway P (1946). The part played by electric fish in the early history of bioelectricity and electrotherapy. Bull Hist Med.

[CR56] Kim AJ, Fitzgerald JK, Maimon G (2015). Cellular evidence for efference copy in *Drosophila* visuomotor processing. Nat Neurosci.

[CR57] Knudsen EI (1982). Auditory and visual maps of space in the optic tectum of the owl. J Neurosci.

[CR58] Knudsen EI, Konishi M (1978). A neural map of auditory space in the owl. Science.

[CR59] Knudsen EI, Konishi M (1979). Mechanisms of sound localization in the barn owl (*Tyto alba*). J Comp Physiol.

[CR60] Knudsen EI, Konishi M, Pettigrew JD (1977). Receptive fields of auditory neurons in the owl. Science.

[CR61] Knudsen EI, Blasdel GG, Konishi M (1979). Sound localization by the barn owl (*Tyto alba*) measured with the search coil technique. J Comp Physiol.

[CR62] Konishi M (1993). Neuroethology of sound localization in the owl. J Comp Physiol A.

[CR63] Köppl C (1997). Phase locking to high frequencies in the auditory nerve and cochlear nucleus magnocellularis of the barn owl, *Tyto alba*. J Neurosci.

[CR64] Köppl C, Gleich O, Manley G (1993). An auditory fovea in the barn owl cochlea. J Comp Physiol A.

[CR65] Krahe R, Maler L (2014). Neural maps in the electrosensory system of weakly electric fish. Curr Opin Neurobiol.

[CR66] Krapp H, Hengstenberg R (1996). Estimation of self-motion by optic flow processing in single visual interneurons. Nature.

[CR67] Krogh A (1929). The progress of physiology. Am J Physiol.

[CR68] Land MF, Collett TS (1974). Chasing behaviour of houseflies (*Fannia canicularis*). J Comp Physiol.

[CR69] Lazzaro J, Mead CA (1989). A silicon model of auditory localization. Neural Comput.

[CR70] Leonhardt A, Ammer G, Meier M, Serbe E, Bahl A, Borst A (2016). Asymmetry of *Drosophila* ON and OFF motion detectors enhances real-world velocity estimation. Nat Neurosci.

[CR71] Lissmann H (1951). Continuous electrical signals from the tail of a fish *Gymnarchus niloticus Cuv*. Nature.

[CR72] Lissmann HW (1958). On the function and evolution of electric organs in fish. J Exp Biol.

[CR73] Mané-Damas M, Molenaar PC, Ulrichts P, Marcuse F, De Baets MH, Martinez-Martinez P, Losen M (2022). Novel treatment strategies for acetylcholine receptor antibody-positive myasthenia gravis and related disorders. Autoimmun Rev.

[CR74] Manger PR, Cort C, Ebrahim N, Goodman A, Henning J, Karolia M, Rodrigues SL, Štrkalj G (2008). Is 21st century neuroscience too focused on the rat/mouse model of brain function and dysfunction?. Front Neuroanat.

[CR75] Marr D (1982). Vision.

[CR76] Mauss AS, Borst A (2020). Optic flow-based course control in insects. Curr Opin Neurobiol.

[CR77] Mazer JA (1998). How the owl resolves auditory coding ambiguity. Proc Natl Acad Sci USA.

[CR78] Moiseff (1989). Binaural disparity cues available to the barn owl for sound localization. J Comp Physiol A.

[CR79] Moiseff (1989). Bi-coordinate sound localization by the barn owl. J Comp Physiol A.

[CR80] Namiki S, Ros IG, Morrow C, Rowell WJ, Card GM, Korff W, Dickinson MH (2022). A population of descending neurons that regulates the flight motor of *Drosophila*. Curr Biol.

[CR81] Neveln ID, Bai Y, Snyder JB, Solberg JR, Curet OM, Lynch KM, MacIver MA (2013). Biomimetic and bio-inspired robotics in electric fish research. J Exp Biol.

[CR82] Nicholas S, Leibbrandt R, Nordström K (2020). Visual motion sensitivity in descending neurons in the hoverfly. J Comp Physiol A.

[CR83] Oram TB, Card GM (2022). Context-dependent control of behavior in *Drosophila*. Curr Opin Neurobiol.

[CR84] Payne R (1971). Acoustic localization of prey by barn owls (*Tyto alba*). J Exp Biol.

[CR85] Pedraja F, Herzog H, Engelmann J, Jung SN (2021). The use of supervised learning models in studying agonistic behavior and communication in weakly electric fish. Front Behav Neurosci.

[CR86] Peña JL, Konishi M (2001). Auditory spatial receptive fields created by multiplication. Science.

[CR87] Pitchers WR, Constantinou SJ, Losilla M, Gallant GR (2016). Progress, prospects, and new tools for neuroethology. J Physiol Paris.

[CR88] Rasnow B (1996). The effects of simple objects on the electric field of *Apteronotus*. J Comp Physiol A.

[CR89] Reichardt W (1987). Evaluation of optical motion information by movement detectors. J Comp Physiol A.

[CR90] Riehle A, Franceschini N (1984). Motion detection in flies: parametric control over ON–OFF pathways. Exp Brain Res.

[CR91] Roulin A (2020). Barn owl: evolution and ecology.

[CR92] Ryu L, Kim SY, Kim AJ (2022). From photons to behaviors: neural implementations of visual behaviors in *Drosophila*. Front Neurosci.

[CR93] Schillberg P, Brill S, Nikolay P, Ferger R, Gerhard M, Führ H, Wagner (2020). Sound localization in barn owls studied with manipulated head-related transfer functions: beyond broadband interaural time and level differences. J Comp Physiol A.

[CR94] Shadron K, Peña JL (2023). Development of frequency tuning shaped by spatial cue reliability in the barn owl's auditory midbrain. Elife.

[CR95] Shinomiya K, Nern A, Meinertzhagen IA, Plaza SM, Reiser MB (2022). Neuronal circuits integrating visual motion information in *Drosophila melanogaster*. Curr Biol.

[CR96] Shumway C (1989). Multiple electrosensory maps in the medulla of weakly electric gymnotiform fish. I. Physiological differences. J Neurosci.

[CR97] Silies M, Gohl DM, Fisher YE, Freifeld L, Clark DA, Clandinin TR (2013). Modular use of peripheral input channels tunes motion-detecting circuitry. Neuron.

[CR98] Silva Barbato AC, Zubizarreta L, Quintana L (2020). A teleost fish model to understand hormonal mechanisms of non-breeding territorial behavior. Front Endocr.

[CR99] Stacho M, Herold C, Rook N, Wagner H, Axer M, Amunts K, Güntürkün O (2020). A cortex-like canonical circuit in the avian forebrain. Science.

[CR100] Takahashi T, Konishi M (1986). Selectivity for interaural time difference in the owl's midbrain. J Neurosci.

[CR101] Thyselius M, Ogawa Y, Leibbrandt R, Wardill TJ, Gonzalez-Bellido PT, Nordström K (2023). Hoverfly *(Eristalis tenax*) pursuit of artificial targets. J Exp Biol.

[CR102] von der Emde G (2007). Active electrolocation of weakly electric fish as a model for active sensing in technical systems. J Bionic Eng.

[CR103] Vonderschen K, Wagner H (2014). Detecting interaural time differences and remodeling their representation. Trends Neurosci.

[CR104] Wagner H, Takahashi T, Konishi M (1987). Representation of interaural time difference in the central nucleus of the barn owl's inferior colliculus. J Neurosci.

[CR105] Wagner H, Weger M, Klaas M, Schröder W (2017). Features of owl wings that promote silent flight. Interface Focus.

[CR106] Watanabe A, Takeda K (1963). The change of discharge frequency by A.C. stimulus in a weak electric fish. J Exp Biol.

[CR107] Waxman SG (2002). Sodium channels as molecular targets in multiple sclerosis. J Rehabil Res Dev.

[CR108] Worm M, Landgraf T, von der Emde G (2021). Electric signal synchronization as a behavioural strategy to generate social attention in small groups of mormyrid weakly electric fish and a mobile fish robot. Biol Cybern.

[CR109] Yang HH, Clandinin TR (2018). Elementary motion detection in *Drosophila*: algorithms and mechanisms. Annu Rev vis Sci.

[CR110] Zakon HH, Lu Y, Zwickl D, Hillis D (2006). Sodium channel genes and the evolution of diversity in communication signals of electric fishes: Convergent molecular evolution. Proc Natl Acad Sci USA.

[CR111] Zakon HH, Zwickl DJ, Hillis DM (2008). Molecular evolution of communication signals in electric fish. J Exp Biol.

[CR112] Zeil J (1983). Sexual dimorphism in the visual system of flies: the compound eyes and neural superposition in Bibionidae (Diptera). J Comp Physiol.

[CR113] Zeil J (1983). Sexual dimorphism in the visual system of flies: the free flight behaviour of male Bibionidae (Diptera). J Comp Physiol.

[CR114] Zupanc GKH (2006). Neurogenesis and neuronal regeneration in the adult fish brain. J Comp Physiol A.

[CR115] Zupanc GKH, Rössler W (2022). Government funding of research beyond biomedicine: challenges and opportunities for neuroethology. J Comp Physiol A.

