# Supplementary material for: Model organisms and systems in neuroethology: one hundred years of history and a look into the future
Source: J Comp Physiol A Neuroethol Sens Neural Behav Physiol. 2024 Jan 16;210(2):227–42. doi: 10.1007/s00359-023-01685-z (PMC10995084; doi:10.1007/s00359-023-01685-z)
Supplement: Supplementary file 1 — Supplementary file1 (DOCX 12 KB) [file 359_2023_1685_MOESM1_ESM.docx]

Table S1: Number of articles published in the decades

| 1924-1933 | 1934-1943 | 1948-1953* | 1954-1963 | 1964-1973 | 1974-1983 | 1984-1993 | 1994-2003 | 2004-2013 | 2014-2023 |
| --- | --- | --- | --- | --- | --- | --- | --- | --- | --- |
| 552 | 341 | 146 | 375 | 1035 | 2310 | 1588 | 1279 | 1004 | 752 |

* note that no articles were published between 1944 and 1947
