# Supplementary material for: Model organisms and systems in neuroethology: one hundred years of history and a look into the future
Source: J Comp Physiol A Neuroethol Sens Neural Behav Physiol. 2024 Jan 16;210(2):227–42. doi: 10.1007/s00359-023-01685-z (PMC10995084; doi:10.1007/s00359-023-01685-z)
Supplement: Supplementary file 2 — Supplementary file2 (DOCX 12 KB) [file 359_2023_1685_MOESM2_ESM.docx]

Table S2: Time to first number of publications

| # | 1000 | 2000 | 3000 | 4000 | 5000 | 6000 | 7000 | 8000 | 9000 |
| --- | --- | --- | --- | --- | --- | --- | --- | --- | --- |
| year | 1952 | 1970 | 1976 | 1980 | 1985 | 1991 | 1998 | 2007 | 2018 |
| years | 28 | 18 | 6 | 4 | 5 | 6 | 7 | 9 | 11 |
