# Supplementary material for: Model organisms and systems in neuroethology: one hundred years of history and a look into the future
Source: J Comp Physiol A Neuroethol Sens Neural Behav Physiol. 2024 Jan 16;210(2):227–42. doi: 10.1007/s00359-023-01685-z (PMC10995084; doi:10.1007/s00359-023-01685-z)
Supplement: Supplementary file 3 — Supplementary file3 (DOCX 14 KB) [file 359_2023_1685_MOESM3_ESM.docx]

Table S3 Number of publications per taxon

| # pub-lica-tions/  taxon | # of taxa | % re: 1512 taxa | % cumu-lative | % publi-cations re: 6751* |  | # pub-lica-tions/  taxon | # of taxa | % | % cumu-lative | % publi-cations |
| --- | --- | --- | --- | --- | --- | --- | --- | --- | --- | --- |
| 1 | 873 | 57.74 | 57.74 | 12.93 |  | 28 | 4 | 0.26 | 97.75 | 1.54 |
| 2 | 248 | 16.40 | 74.14 | 7.35 |  | 32 | 7 | 0.46 | 98.21 | 3.11 |
| 3 | 109 | 7.21 | 81.35 | 4.84 |  | 36 | 2 | 0.13 | 98.35 | 1.01 |
| 4 | 67 | 4.43 | 85.78 | 3.97 |  | 40 | 3 | 0.20 | 98.54 | 1.69 |
| 5 | 42 | 2.78 | 88.56 | 3.11 |  | 50 | 6 | 0.40 | 98.94 | 4.00 |
| 6 | 24 | 1.59 | 90.15 | 2.13 |  | 60 | 1 | 0.07 | 99.01 | 0.81 |
| 7 | 17 | 1.12 | 91.27 | 1.76 |  | 70 | 3 | 0.20 | 99.21 | 2.89 |
| 8 | 22 | 1.46 | 92.72 | 2.61 |  | 80 | 3 | 0.20 | 99.40 | 3.33 |
| 9 | 10 | 0.66 | 93.39 | 1.33 |  | 90 | 0 | 0 | 99.40 | 0. |
| 10 | 12 | 0.79 | 94.18 | 1.78 |  | 100 | 0 | 0 | 99.40 | 0. |
| 12 | 14 | 0.93 | 95.11 | 2.28 |  | 150 | 3 | 0.20 | 99.60 | 5.55 |
| 14 | 3 | 0.20 | 95.30 | 0.58 |  | 200 | 4 | 0.26 | 99.87 | 10.37 |
| 16 | 12 | 0.79 | 96.10 | 2.67 |  | 300 | 1 | 0.07 | 99.93 | 3.70 |
| 18 | 4 | 0.26 | 96.36 | 1.01 |  | 400 | 0 | 0 | 99.93 | 0 |
| 20 | 6 | 0.40 | 96.76 | 1.69 |  | 500 | 0 | 0 | 99.93 | 0 |
| 24 | 11 | 0.73 | 97.49 | 3.58 |  | 600 | 1 | 0.07 | 100 | 8.15 |

#: number; *we lost references if the taxon was not clearly stated. For example, we did not count "fly" in the %publications, because the term is too general. We also did not include taxa where only the genus was mentioned, if there were two species with the same genus name already included.
