# Supplementary material for: Model organisms and systems in neuroethology: one hundred years of history and a look into the future
Source: J Comp Physiol A Neuroethol Sens Neural Behav Physiol. 2024 Jan 16;210(2):227–42. doi: 10.1007/s00359-023-01685-z (PMC10995084; doi:10.1007/s00359-023-01685-z)
Supplement: Supplementary file 4 — Supplementary file4 (DOCX 13 KB) [file 359_2023_1685_MOESM4_ESM.docx]

Table S4: Taxa with more than 100 publications in the journal

| Name | number | Comment* |
| --- | --- | --- |
| Bee | 524 | Honey bee (45)^$^, honey-bee+honeybee (182), Biene (179), *A. mellifica* (35), *A. mellifera* (83) |
| Locust | 274 | Locust (202), *Locusta* (3), *Locusta migratoria* (69) |
| Crayfish^§^ | 200 | Crayfish (166), *Procambarus* (5), *Procambarus clarkii* (29) |
| Fruitfly | 175 | fruitfly (2), *Drosophila* (103), *Drosophila melanogaster* (70) |
| Cricket | 165 | Grille (8), cricket (102), *Gryllus bimaculatus* (55) |
| Cockroach | 152 | Cockroach (96), Schabe (1), *Periplaneta americana* (55) |
| Blowfly^§^ | 134 | Schmeissfliege (1), blowfly (43), *Calliphora* (42); *Calliphora* *erythrocephala* (48) |
| Pigeon | 120 | *Columba livia*+Taube (25), pigeon (93), Columbides (french) (2) |
| Medicinal leech | 108 | Egel (4), Blutegel (3), leech(47), medicinal leech (31), *Hirudo medicinalis* (23) |

* We were careful not to have double-counts despite overlapping names, ^$^numbers for specific names in brackets, ^§^numbers are a bit unclear due to the unspecific common name
