# Supplementary material for: Model organisms and systems in neuroethology: one hundred years of history and a look into the future
Source: J Comp Physiol A Neuroethol Sens Neural Behav Physiol. 2024 Jan 16;210(2):227–42. doi: 10.1007/s00359-023-01685-z (PMC10995084; doi:10.1007/s00359-023-01685-z)
Supplement: Supplementary file 5 — Supplementary file5 (DOCX 14 KB) [file 359_2023_1685_MOESM5_ESM.docx]

Table S5: Representation of major current model systems and human research in the journal

| *Drosophila melanogaster* | 175 publications from 1932 to 2020 |
| --- | --- |
| *Aplysia* spp. | 87 publications from 1932 to 2022 |
| *Mus musculus* (Mouse) | 68 publications from 1929 to 2019 |
| Non-human Primates | 51 publications from 1926 to 2021 |
| *Homo sapiens* (Human) | 20 publications from 1930 to 2020 |
| *Caenorhabditis elegans* | 8 publications from 1980 to 2020 |
| *Danio rerio* (Zebrafish) | 2 publications: 1995, 2012 |
